# Pyloric Stenosis in Infants

**Published:** 1907-09-21

**Authors:** 


### Diseases of Children.

### PYLORIC STENOSIS IN INFANTS.

There is perhaps no pathological condition of early life which has received so much attention of late as that of stenosis of the pylorus. Its cause is still much disputed. Two theories, however, have been put forward to account for its origin. One maintains that the stenosis is the result of actual pyloric hypertrophy, of a primary nature and possibly even of intrauterine development. The other theory is that the hypertrophy is merely secondary to spasm. The question, however, is still an open one as to how this spasm is originated. It might possibly be due to improper feeding, but against this view is the fact that pyloric stenosis may occur in infants who are correctly fed in every respect. It may be that the spasm is due to some defect in the gastric movements, but what this defect is, or how it is brought about, cannot as yet be satisfactorily explained.

The age at which the symptoms are first observed varies in different cases. Most patients come under observation during the first month, while practically none are seen for the first time after the second month. Generally speaking, the infant is born perfectly healthy. Males appear to be more liable to this condition than females. The leading symptom is vomiting. This is usually of a projectile nature, and often comes on immediately after feeding, but does not necessarily occur after every feed. The patient may vomit up accumulations of partially digested milk. Constipation is an important symptom. It is the result of non-passage of stomach contents into the intestine, and, when combined with vomiting, is always significant. In many cases the peristaltic movements of the stomach are visible, especially if the abdominal walls are gently stroked by the fingers. In certain cases, too, an actual thickening of the pyloric ring can be made out, but this is by no means always present. A greater or less degree of general malnutrition is naturally always to be observed.

### The Diagnosis.

The diagnosis of pyloric stenosis is not always an easy matter. It can only be definitely made in most cases after prolonged observation. In the first place we must make sure that the symptoms are not due to improper feeding; in the second place, that they are not the result of simple spasm of the pylorus; and, lastly, that they are not due to some other condition altogether. Thus a German observer recently recorded the case of an infant who presented all the symptoms of pyloric stenosis, and yet after death was discovered to have a duodenal ulcer. Probably no case can be definitely said to be one of stenosis in which some thickening of the pylorus cannot be made out after repeated examinations. (A single examination may fail to detect it.) Where there are vomiting, constipation, and visible peristalsis, a diagnosis of pylorospasm may be made, and if after suitable treatment the patient fails to respond, and especially if the infant continues to lose weight, then we are probably warranted in regarding the case as one of actual stenosis of the pylorus. The projectile character of the vomiting is a diagnostic point of considerable value, apart from the other symptoms, but of itself is not by any means sufficient.

### Treatment.

The treatment is usually stated to be medical and surgical. When medical means fail, operation is seldom of any real value. At first the principal object aimed at is to get rid of any irritating material present in the stomach. For this purpose nothing is so effectual as daily lavage. This is best done by using plain boiled water; in some cases the present writer has found the addition of two teaspoonfuls of bicarbonate of sodium to the pint of water serviceable in dissolving and getting rid of the excess of mucus which is invariably present. Lavage ought to be carried out persistently every day for weeks if success is to be looked for. The feeding of the patient is also of the greatest importance. Breast milk is best, and there is no really good substitute for it. It is well to remember the value of breast milk, as most of these cases have been taken off the breast under the belief that the "milk did not suit." Failing the breast, the infant may be put on condensed milk or on whey. Sometimes peptonised milk has been found successful when other things have failed. The main essentials in feeding the patient are the size and frequency of the meals and their low proteid and fat constitution. It is advisable to begin with ounce feeds every hour or every hour and a half. Whey is of great value, because it is less rich in proteid than milk; but it must be carefully prepared, otherwise it becomes very distasteful. A case recently under our care was absolutely cured by lavage and whey feeding, and the administration of bicarbonate of sodium, which is, we believe, a useful addition to the treatment of such cases.

### Drugs and Surgery.

Regarding drugs, little need be said. It may be thought advisable to administer something in order to overcome the element of spasm which must be present in every case. For this purpose opium, belladonna, and sodium bromide are indicated. When these are employed they should be prescribed in small but often-repeated doses.

Up to the present no remarkable success has attended the surgeon in dealing with this condition. Too often the result of surgical interference is the death of the patient, and at the present time the pendulum seems to be swinging to the medical treatment of pyloric stenosis. After all, we must not forget that in all probability the majority of cases presenting symptoms of the condition under discussion are simply examples of pylorospasm which are bound to recover under prompt and assiduous medical treatment. Cases of actual stenosis of the pylorus are much rarer, and even these may be quite amenable to the therapeutic measures we have here outlined.

